# Predictors of Delay in Seeking Health Care among Myocardial Infarction Patients, Minia District, Egypt

**DOI:** 10.1155/2015/342361

**Published:** 2015-12-08

**Authors:** Eman Ramadan Ghazawy, Amany Edward Seedhom, Eman Mohamed Mahfouz

**Affiliations:** Public Health Department, Faculty of Medicine, Minia University, Minia 61111, Egypt

## Abstract

*Objectives*. To determine the barriers that hinder early seeking of medical care among Minia's myocardial infarction patients.* Methods*. The study was based on individual interviews with 207 men and women with a first confirmed myocardial infarction (MI), admitted to the coronary care units of hospitals in Minia city in the period from April 1 to August 30, 2014. Data was collected via structured questionnaire and patient medical charts. The delay was evaluated by assisting patients to triangulate time of symptom onset and time of professional health care by placing both times in context of daily activities that participants could easily remember.* Results*. The median (25th, 75th percentiles) delay time was 4 (2, 10) h. Only 32.8% of patients arrived within 2 hours of symptoms onset. Variables that significantly predicted prehospital delay time were patient's misinterpretation of nature of pain with OR 8.98 (95% CI) (3.97–20.32), illiteracy 7.98 (2.77–22.95), age (>65) 5.07 (1.57–16.29), and pain resistance behavior 4.61 (2.04–10.41).* Conclusions*. Interventions to decrease prehospital delay must focus on improving public awareness of acute myocardial infarction symptoms and increasing their knowledge on early treatment benefits.

## 1. Introduction

Coronary artery disease (CAD) is the most common cause of morbidity and mortality worldwide [1]. Being a life threatening manifestation of CAD, acute myocardial infarction (AMI) needs prompt recognition and management. Approximately one-third of deaths from AMI occur within few hours of onset of symptoms and usually before the patients reach to hospital [2].

Although AMI is associated with relatively high morbidity and mortality, it is well known that timely reperfusion therapy can result in dramatically improved patient clinical outcomes [3–5].

Delays in seeking medical care are associated with adverse consequences on patients' conditions and medical cost and limit the potential benefits of early interventions [6, 7]. The quotation “time is muscle” is used to highlight the importance of saving time and starting treatments without delay [8, 9].

Early thrombolytic therapy improves perfusion of myocardial ischemic area, limits infarct size, and reduces risk of fatal arrhythmias. It increases survival rate up to 50% when provided within one hour after symptom onset. However, many patients with AMI do not benefit because of seeking medical care late, as around 50% seek medical care after 2 hours and more than one-quarter with AMI are referred to the medical center after 6 hours. Factors associated with prolonged prehospital delay in patients with AMI have been the subject of interest in various studies. Many factors such as being old [10], being female [11], having low socioeconomic status, and being Black [12], clinical factors such as a history of hypertension or diabetes, or prior history of angina or previous AMI, and other factors such as consultation with one's spouse, family member, or physician [13] have been associated with longer delay.

However, the majority of these studies were concerned with western populations. Factors associated with prolonged prehospital delay might differ among populations due to diversity in ethnicity, culture, socioeconomic status, health care system organization, and so forth. Recognition of the contributing factors may help to find and develop new interventions to lessen delays and AMI morbidity and mortality rate.

There is no enough information on the effective factors of delay time in AMI in Minia. Therefore, the aim of the present study was to determine the length of delay and investigate the causes of delay in seeking treatment among Minia patients with AMI.

## 2. Methodology

This cross-sectional study was conducted among all patients with confirmed first acute myocardial infarction (AMI) admitted to the coronary care units of hospitals in Minia city in the period from April 1 to August 30, 2014.

210 patients were interviewed. Patients were pain-free and hemodynamically stable at the time of interview. Individuals diagnosed with the new diagnostic criteria for AMI [14] were eligible for inclusion in the study with no restriction on age. Patients who had a cardiac arrest before admission and those who had cognitive disorders were excluded from the study. Three patients were excluded due to the lack of reliable data.

Total prehospital delay (TPD) was the time (in minutes) from symptom onset to arrival at the emergency department.

The questionnaire contained demographic characteristics of patients, history of medical problems, clinical manifestation of patients at the admission time, sociocultural factors related to delay, and first patient's reactions to MI symptoms. To determine the reliability, a pilot study was conducted among 50 patients and the reliability of this questionnaire was approved using Cronbach's alpha test (*α* = 0.87). It took 15 minutes to fill in the questionnaires through bedside interviews.

The delay was evaluated by assisting patients to triangulate time of symptom onset and time of professional health care by placing both times in context of daily activities that participants could easily remember.

### 2.1. Statistical Analysis

Data entry and analysis were all done by using software SPSS (Statistical Package for the Social Sciences) version 19. Data were expressed as frequency and percentage, mean ± standard deviation, or median where appropriate. Differences in the distribution of characteristics of the patients, classified according to the extent of prehospital delay, were examined using the chi-square test for the discrete variables. Logistic regression analysis was utilized to determine which of the sociodemographic, clinical, and behavioral characteristics best distinguished between patients with delay and those without. The probability of less than 0.05 was used as a cutoff point for all significant tests.

## 3. Results

### 3.1. Baseline and Demographic Characteristics of the Participants

This study included 207 patients diagnosed with AMI. The comparison of prehospital delay times by sociodemographic and clinical characteristics of the patients was presented in Table 1. The overall median (25th, 75th percentiles) delay time was 4 (2, 10) h. Of the study group, 32.4% arrived at hospital within 2 hours after the onset of symptoms.

Among patients, those who delayed seeking medical care for >2 hours, it was found that 31.4% of them were aged patients (>65 years old) and more than half (55%) were from rural areas compared to 10.5% and 35.8% of those who arrived at the hospital within 2 hours, respectively, and these differences were statistically significant (*p* value < 0.0001 and 0.01), respectively. Regarding educational and occupational status, it was found that there were statistically significant differences between patients who delayed seeking medical care and those who did not; 46.4% were illiterates and 47.2% unemployed, compared to 14.9% and 23.9%, respectively. Gender and marital status showed no statistically significant differences with prehospital delay.

Concerning clinical characteristics, 20.7% of patients with prehospital delay >2 hours had history of previous angina compared to 38.8% of those who arrived at the hospital within 2 hours (*p* = 0.006). There was no statistically significant difference between both categories regarding diabetes and hypertension (Table 1).

### 3.2. Perception of Pain When It Occurs

Regarding the interpretation of the nature of pain, 23.7% of the participants associated it with a heart problem. The remaining thought of other causes for the pain, interpreting it as a temporary discomfort, a stomach problem, anger, cramps, and more, or even did not imagine what that was.

About 67% of patients showed pain resistance behavior; we observe that these behaviors are expressed by actions which are attempts to mitigate (e.g., sipping a glass of milk, taking medicine, going to sleep, taking sugar water, and massaging the chest); some patients try to bear and hide the pain (e.g., keeping quiet, not speaking, and not mentioning the pain), and others hope for it to improve and continue activities even with pain (Table 2).

### 3.3. Causes of Prehospital Delay

Some attitudes were found to contribute to patients' delay in deciding to seek medical attention, 39.3% of delayed patients did not consider the symptoms to be serious, 10% found it unpleasant or embarrassing to seek medical help, and 5.7% did not want to be a burden on anyone.

Other causes related to some contextual factors; as in the patients' opinions, the most common cause of prehospital delay was lack of equipment and proper first line medications, as approximately 25.7% went to doctors' offices, health centers, and clinics or were seen by a doctor at home, live farther from hospital (13.6%), and lack suitable transportation (5.7%) (Table 3).

### 3.4. Factors Related to Prolonged Prehospital Delay

In multivariate regression analysis (Table 4), it was found that patient's misinterpretation of the nature of pain with OR (95% CI), 8.98 (3.97–20.32), illiteracy, 7.98 (2.77–22.95), age (>65 years), 5.07 (1.57–16.29), and pain resistance behavior, 4.61 (2.04–10.41), were significant predictors of prolonged prehospital delay.

## 4. Discussion

In this study, the median delay time was 4 hours; this median time is higher than that calculated in a previous study [15] and lower than in another [16]. Two studies showed that a median of prehospital delay in four hospitals in London was 2 hours and in five hospitals in the USA this time was 4.25 hours [15, 16].

In the current study it was found that 73.4% of patients reported that this was the first episode of AMI; this result was consistent with the findings of Mussi et al. [17], who studied the time of decision for seeking medical care and the time of arrival at a health facility for patients with AMI in Salvador and found that vast majority reported that this was the first attack of AMI. This fact may explain why only 23.7% of the participants associated the pain with a heart problem.

The patients' ability to interpret the symptoms correctly decisively determines their behavior. Our finding confirmed that the majority (88.6%) of patients who delayed seeking medical care for >2 hours misinterpret the nature of symptoms, and 71.4% showed pain resistant behaviors. These results were consistent with the findings of Mussi et al. [17], who found that participants who interpreted the pain as cardiovascular in nature took much less time to decide to seek medical care and to reach a health facility and those who expressed pain resistance behaviors took longer time in both to decide to seek medical care and to reach a health facility.

Additionally, the crude OR showed that patients who misinterpreted the nature of symptoms and expressed pain resistance behaviors had about 7- and 2-fold, respectively, increases for delay in seeking help more than those who attributed their symptoms to a cardiac problem and those who did not show pain resistance behaviors (Table 2). Instantly, after adjustment for other factors it showed that both variables were significant predictors in prehospital delay (*p* < 0.0001), with an OR = 8.98 (3.97–20.32) and 4.61 (2.04–10.41), respectively. Such results were in accordance with that of Fukuoka et al. (2005) [18] who conducted a study to examine whether culture is associated with a delay in accessing medical care in Japanese patients experiencing symptoms of AMI and found that symptom interpretation on the part of the patients accounted for the largest unique contribution among significant independent variables in prehospital delay time.

A number of sociodemographic, clinical, and behavioral factors have been associated with late presentation at hospital for treatment after the onset of acute coronary symptoms. Based on our results, older people (>65 years old) delayed seeking medical care compared to younger ones. This finding was in agreement with previous published reports declaring that older people experienced longer delay in seeking medical attention compared to younger ones [19–21]. This could be due to older people having limited access to medical care, especially when they live alone. Furthermore, elderly people often have many concomitant diseases that can be regarded as causes of discomfort. Previous investigators have reported conflicting findings about delay times between men and women. Some investigators [22–24] found that women delayed longer than men, while others found no significant differences. We documented no significant differences in delay times between men and women in this current study.

Regarding educational level, illiteracy was found to be a significant predictor for prehospital delay in both univariate and multivariate analysis. This was in accordance with Farshidi et al. [25] who conducted a study to evaluate the factors associated with prehospital delay in patients with AMI in Iran and found that illiterate patients had a higher rate of prehospital delay. Sari and his colleagues (2008) [26], who investigated the factors associated with prolonged prehospital delay in patients with AMI in Turkey, found that total education time of less than nine years was an independent predictor of prolonged prehospital delay OR = 2.27 (1.42–3.60). We hypothesized that a high level of education may allow better awareness of symptoms and better knowledge of myocardial infarction and therefore reduce symptom-to-first-medical-contact time, thus explaining our results.

In the current study, we found that employment status was significantly associated with prehospital delay; 47.2% unemployed patients delayed seeking medical care. This could be explained by the high cost of health care services as the unemployed persons were not covered by governmental health insurance and it could be due to old age because in our sample 41.5% of the unemployed were >65 years old.

In this study causes of prehospital delay were investigated from the patients' point of view. Emotional attitudes were found to be important determinants of patient delay in acute myocardial infarction. The findings of the current study were consistent with Leslie et al. [27] who studied the reasons for the delay and subsequent pattern of accessing care among 313 surviving cases from the Glasgow MONICA (monitoring trends and determinants in cardiovascular disease) coronary event register and found that the most frequently given reason was “thinking that the symptoms would go away” and “not thinking it was serious.” And also our findings are in accordance with Kentsch et al. [28] who identified the following independent contributors to a late decision to seek medical help: wanting to wait and see, not taking symptoms seriously, and not wanting to bother anybody.

## 5. Limitations of the Study

Data are dependent on the accuracy and completeness of the individual hospital data abstraction and original chart documentation.

## 6. Conclusion and Recommendations

The process of decision-making regarding whether to seek medical assistance following the onset of chest pain that could be due to a heart attack is multifactorial requiring multifaceted complex interventions. Important elements to address in any intervention seem to be increased awareness about symptoms of AMI, perceived seriousness of the symptoms, and the process of coping with the symptoms adopted. A program of health education about symptoms of AMI and the importance of early seeking care should be conducted through mass media. The authors advise the cardiologists to distribute a brochure about 2nd prevention to patients after their recovery.

## Figures and Tables

**Table 1:** Prehospital delay in relation to baseline and demographic characteristics of the participants.

Variables	All (*n* = 207)	Early arrival ≤ 2 hours (*n* = 67) = 32.3%	Prehospital delay > 2 hours (*n* = 140) 67.7%	*χ* ^2^	OR (95% CI)	*p* value
Age						
<50 years old	49 (23.7)	26 (38.8)	23 (16.4)	17.69	Reference	0.0001^*∗*^
50–65 years old	107 (52.7)	34 (50.7)	73 (52.1)		2.43 (1.21–4.85)	
>65 years old	51 (24.6)	7 (10.5)	44 (31.4)		7.11 (2.68–18.84)	
Sex						
Male	155 (74.9)	55 (82.1)	100 (71.4)	2.73	Reference	0.09
Females	52 (25.1)	12 (17.9)	40 (28.6)		1.83 (0.88–3.78)	
Residence						
Urban	106 (51.2)	43 (64.2)	63 (45)	6.67	Reference	0.01^*∗*^
Rural	101 (48.8)	24 (35.8)	77 (55)		2.19 (1.20–3.99)	
Marital status						
Married	164 (79.3)	57 (85.1)	107 (76.4)	2.05	Reference	0.2
Unmarried (widow, single, or divorced)	43 (20.7)	10 (14.9)	33 (23.6)		1.75 (0.81–3.82)	
Educational level						
University/above	58 (28.1)	29 (43.3)	29 (20.7)	21.66	Reference	0.0001^*∗*^
Below university	74 (35.7)	28 (41.8)	46 (32.9)		1.6 (0.82–3.29)	
Illiterate	75 (36.2)	10 (14.9)	65 (46.4)		6.50 (2.80–15.07)	
Occupation						
Professional	43 (20.8)	21 (31.3)	22 (15.7)	12.51	Reference	0.006^*∗*^
Cleric	35 (16.9)	14 (20.9)	21 (15)		1.8 (0.79–4.32)	
Manual working	47 (22.7)	16 (23.9)	31 (22.1)		1.4 (0.58–3.53)	
Unemployed	82 (39.6)	16 (23.9)	66 (47.2)		3.9 (1.75–8.85)	
Risk factors						
Hypertension						
Yes	105 (50.7)	38 (56.7)	67 (47.9)	1.42	0.70 (0.39–1.25)	0.2
No	102 (49.3)	29 (43.3)	73 (52.1)		Reference	
Diabetes						
Yes	98 (47.4)	32 (47.8)	66 (47.1)	0.007	0.97 (0.54–1.74)	0.9
No	109 (52.6)	35 (52.2)	74 (52.9)		Reference	
Previous angina						
Yes	55 (26.6)	26 (38.8)	29 (20.7)	7.60	0.41 (0.21–0.78)	0.006^*∗*^
No	152 (73.4)	41 (61.2)	111 (79.3)		Reference	

^*∗*^Statistically significant. OR (95% CI) is odds ratio (95% confidence interval).

**Table 2:** Prehospital delay in relation to the acute perception of symptoms.

Acute perception of symptoms	All (*n* = 207)	Early arrival ≤ 2 hours (*n* = 67)	Prehospital delay > 2 hours (*n* = 140)	*χ* ^2^	OR (95% CI)	*p* value
Interpretation of the nature of pain						
Associate it to heart problem	49 (23.7)	33 (49.3)	16 (11.4)	35.8	Reference	0.0001^*∗*^
Misinterpret the nature of pain	158 (76.3)	34 (50.7)	124 (88.6)		7.52 (3.71–15.62)	
Reaction during pain occurrence						
Seek medical advice	69 (33.3)	29 (43.3)	40 (28.6)	4.4	Reference	0.03^*∗*^
Pain resistance behavior^*▪*^	138 (66.7)	38 (56.7)	100 (71.4)		1.91 (1.04–3.50)	

^*∗*^Statistically significant. OR (95% CI) is odds ratio (95% confidence interval)

^*▪*^Behaviors are expressed by actions which are attempts to mitigate, bear, and hide the pain, hoping for it to improve and to continue activities even with pain.

**Table 3:** Causes of prehospital delay among study participants.

Causes of prehospital delay	No	Percent
Causes related to participant attitude		
Do not consider the symptoms to be serious	55	39.3
Find it unpleasant or embarrassing to seek medical help	14	10
Do not want to be a burden on anyone	8	5.7

Causes related to surrounding factors		
Lack of equipment and proper first line medications	36	25.7
Living in farther distance from hospital	19	13.6
Lack of suitable transportation	8	5.7

Total	140	100

**Table 4:** Logistic regression for factors influencing prehospital delay for patients with AMI.

Variables	Odds ratio (95% confidence interval)	*p* value
Misinterpret the nature of pain	8.98 (3.97–20.32)	<0.0001
Educational level (illiterate)	7.98 (2.77–22.95)	<0.0001
Age (>65 years old)	5.07 (1.57–16.29)	0.006
Pain resistance behavior	4.61 (2.04–10.41)	<0.0001
